# Spontaneous triplet pregnancy and trap sequence, case report

**DOI:** 10.1186/s12884-019-2484-3

**Published:** 2019-09-05

**Authors:** Engin Yıldırım

**Affiliations:** Department of Gynecology and Obstetrics, Hitit University, Faculty of Medicine, Ulukavak District, İkbalkent Campus, Corum, Turkey

**Keywords:** Triplet pregnancy, TRAP sequence

## Abstract

**Background:**

Spontaneous multiple pregnancies are rare, and the incidence of spontaneous triplet pregnancy is about 1/4000. TRAP (Twin Reversed Arterial Perfusion) sequence has acardiac foetus with non-viable multiple anomalies, and there is a pump foetus which feeds this foetus with placental anastomoses. TRAP sequence phenomena is quite rare in triplet pregnancies.

**Case presentation:**

The patient who applied to our clinic was 30 years old. Monochorionic diamniotic triplet pregnancy was detected by ultrasonographic examination. First amniotic sac had one foetus (Foetus A). Ultrasonographic evaluation of Foetus A revealed gestational age of 31 weeks, adequate amniotic fluid and no fetal structural anomalies. The second amniotic sac contained 2 foetuses and polyhydromnios. Ultrasonic measurements of Foetus B were consistent with 32 weeks gestational age. Color flow doppler indicated Foetus B was the pump foetus. Foetus C was an acardiac foetus with no sonographic visualization of cranium, thoracic organs or extremities, but abdominal circumference consistent with 28 weeks. Pregnancy was followed conservatively and evaluated regularly by ultrasonography twice a week. When prolonged bradycardia was detected in fetus B at 35 4/7 weeks, emergency cesarean section was performed. Two healthy fetuses weighing 2 kg were delivered each with an 8/10 APGAR score (Appearance, Pulse, Eye Insertion, Activity, Respiration).

**Conclusion:**

This case was managed without any invasive procedures and demonstrates that treatment of TRAP sequence cases can be individualized considering clinical conditions, the size of the acardia twin and extent of placental venous anastomoses.

## Background

In recent years, emerging of developments in reproductive techniques, there is an increase in the prevalence of multiple pregnancies. Spontaneous multiple pregnancies are rarely seen. Also, the incidence of spontaneous triple pregnancy is determined approximately 1/7000. Triplet pregnancies are known to cause various pregnancy complications. Some of these complications comprise of fetal structural defects, neurological anomalies, amniotic fluid anomalies, preterm labor, and poor neonatal outcomes. The incidence of monochorionic triplet pregnancies is quite low, which is stated 1/100.000 [1].

TRAP (Twin Reversed Arterial Perfusion) sequence has acardiacfoetus with non-viable multiple anomalies, and there is a pump foetus which feeds this foetus with placental anastomoses. The rate of this syndrome, which is caused by TRAP sequence, is 1/35000 in all pregnancies and 1/100 in monochorionic twin pregnancies [2]. Hypo-oxygenized blood coming via umbilical arteries feeds, although not sufficiently, the acardiac twins’ lower extremity and part of abdomen through common iliac artery and abdominal aorta. When the blood reaches upper extremity and cranium of the acardiac foetus, the oxygen saturation which is already low, decreases even more and it causes insufficient tissue and organ development. While chromosome is generally normal in the pump foetus, chromosomal anomalies might be detected in the acardiac foetus. Mortality of the pump foetus increases depending on heart failure and prematurity caused by polyhydroamniosis.

While TRAP sequence cases in monochorionic twin pregnancies are common in literature, TRAP sequence phenomena is quite rare in triplet pregnancies. This paper presents spontaneous triplet pregnancy accompanied by TRAP sequence case. The course and outcome of this case will increase the experience of TRAP sequence pregnancy.

## Case presentation

The patient was 30 years old when she applied to the clinic and had a history of four gravidas and one para. Her history also included two spontaneous aborts, and her living child was born via spontaneous vaginal delivery. In the medical anamnesis, the patient reported that this pregnancy had occurred spontaneously. No genetic anomaly was found in the family history. Iron replacement was performed during pregnancy. The patient, whose previous follow-ups took place in a private centre, was in her 31st gestational week according to her last menstrual period when she intake to our clinic.

**Fig. 1:**
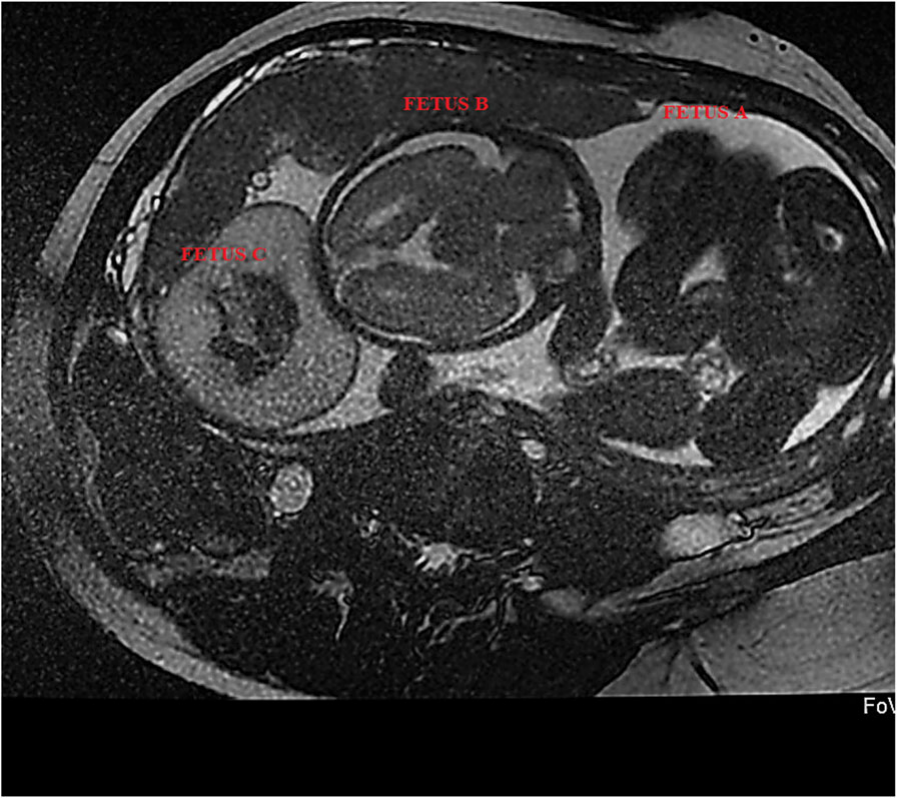
Acardiac fetus image by ultrasonography

Physical examination revealed no significant clinical findings other than third trimester abdominal pregnancy. Our ultrasonographic evaluations indicated a monochorionic diamniotic triplet pregnancy at first visit. Some sonographic markers were used to evaluate chorionicity. V-sign was not observed in ultrasonographic examination. Amniotic membrane thickness was measured less than 1.5 mm. Fetal sexes were female and the sex of the acardiac foetus could not be distinguished. The first amniotic sac contained one foetus (Foetus A). Biometric measurements of this foetus indicated that the foetus was in its 31st gestational week, and amnion liquid in the sac was sufficient. No structural anomalies were detected in the ultrasonographic organ scan of Foetus A. The second amniotic sac included a foetus in its 32nd gestational week (Foetus B) and an acardiac third foetus (Foetus C), as illustrated in Fig. 1. Ultrasonographic evaluations indicated a normal organ scan for Foetus B, but polyhydroamniosis was detected in the amniotic sac. Foetus A was in breech presentation and Foetus B was in head presentation.

**Fig. 2:**
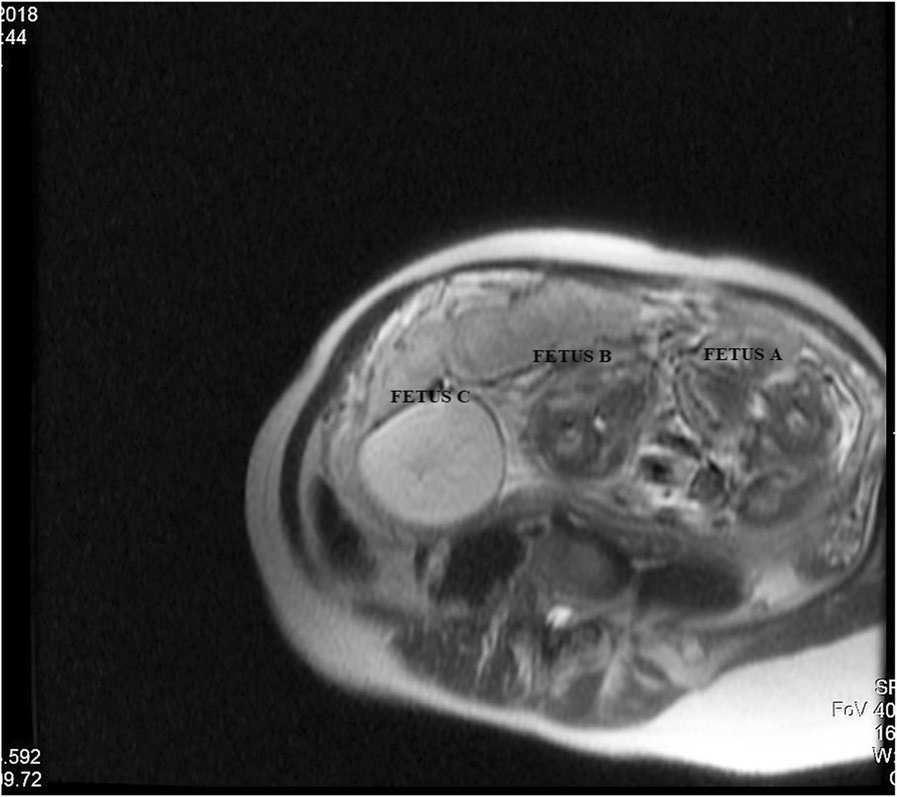
Magnetic resonance view of triplet pregnancy

Ultrasound did not reveal the cranium, thoracic organs and extremities of Foetus C; abdominal circumference was measured at 240.72 mm, indicating that the foetus was at 28 weeks of gestation. The foetus did not have vertebral integrity; and dorsal cystic hygroma, intra-abdominal acid and severe subcutaneous oedema were noted (Fig. 2). The gastric pocket and bladder were not visible, and irregular, dense hyperechogenic areas were detected in the foetal abdomen. An evaluation of Foetus C’s umbilical cord flow showed two arteries and a single vein. The umbilical arterial flow direction was towards to the Foetus C, and the vein flow direction was towards the placenta. Echocardiography was conducted on Foetuses A and B to evaluate cardiothoracic ratio, pericardial effusion, left ventricle output, ventricular ejection force and Tei İndex (myocardial performance index) value, and to conduct colour flow mapping. No cardiomegaly, major vascular anomalies, cardiac output disorders, peak flow velocity disorders or pericardial effusion were detected in Foetuses A and B. Middle cerebral artery Doppler examinations of the healthy foetuses showed that multiple of median (MoM) values ranged between 1,30 and 1,50. A foetal magnetic resonance (MR) assessment was then performed for Foetus C. This examination showed no extremities belonging to Foetus C and indicated clearly that the placenta was monochorionic.

The patient’s non-stress test findings were normal and the pump foetus’s (Foetus B) examination did not indicate cardiac failure; thus, the patient was treated with betamethasone two times. The pregnancy was followed and was evaluated using ultrasound twice per week. Foetuses A and B grew appropriately. The follow-ups, including foetal biometrics, umbilical artery doppler measurements and non-stress testing, were performed six times. Foetus B was found to have improvement in the polyhydramnios detected in the first examination (11.2 cm) and the deepest vertical pocket until birth reached between 8 cm and 10 cm. The amount of amniotic fluid in Foetus A was never below 4 cm in the deepest pocket until birth. The abdominal circumference of Foetus A progressed from the 31st to 34th week, and the abdominal circumference of Foetus B progressed from the 32nd to 35th week. Blood flow in the umbilical and middle cerebral arteries did not worsen.

The patient came for follow-up on the 5th day of the 34th week. Ultrasonographic evaluations indicated bradycardia in the pump foetus (Foetus B). A foetal heart rate ranging between 70 beats per minute (BPM) and 100 BPM lasted approximately 3 min and then was regulated. Due to the bradycardia, an emergency caesarean section was performed. Two live foetuses were delivered via caesarean section, with Foetus A weighing 2010 g and Foetus B weighing 2150 g. The acardiac nonviable foetus (Foetus C) was born weighing about 500 g. The healthy foetuses breathed spontaneously; surviving babies APGAR (Appearance, Pulse, Grimace, Activity, Respiration) scores in the 1st and 5th minutes were recorded as 8/10 and 8/10. The surviving babies were monitored up to 3 months after birth; Foetus A reached 5410 g, and Foetus B reached 5360 g. Transfontanelle and hip ultrasound (Ultrasound Examination for Detection of Developmental Dysplasia of the Hip) examinations were performed for both foetuses and found normal. After birth, babies’echocardiography was repeated twice in one-month intervals and recorded as normal.

**Fig. 3:**
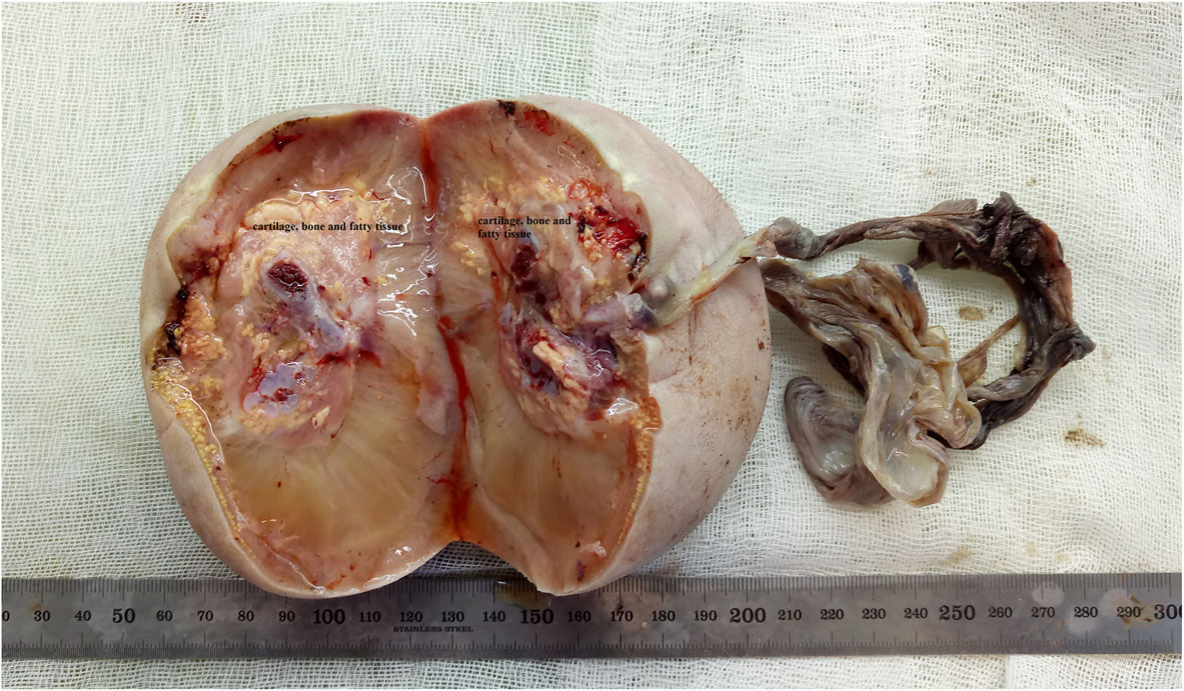
Dissection of acardiac foetus

**Fig. 4:**
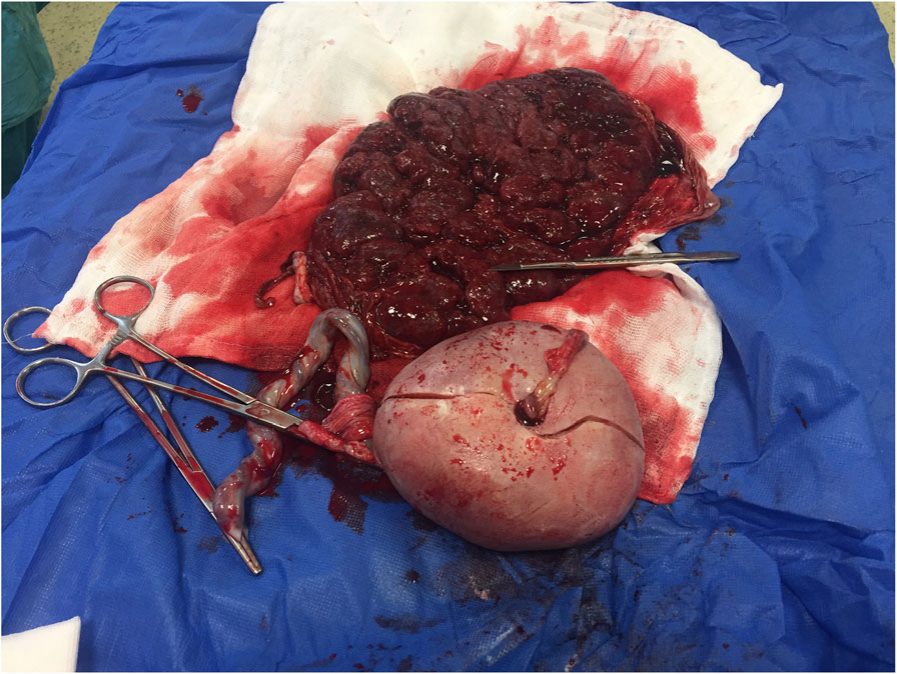
Placental base view

No extremities of the acardiac foetus (Foetus C) were detected in the macroscopic evaluations. Cranium, brain and vertebrae belonging to the central nervous system could not be distinguished. Extensive subcutaneous oedema was observed. Cartilage, bone and fatty tissue areas were present in the tissue dissection. No intra-abdominal organs or thoracic organs could be seen. The pathology of Foetus C indicated an 11 cm × 11 cm × 6.5 cm oval of irregular tissue in the area we thought to be of the abdomen and thorax. The umbilical cord had a length of 2.5 cm and three vein structures. Bone tissue, which was considered to be vertebra, was detected irregularly. Skin and subcutaneous oedema were found. Under subcutaneous dissection, fat tissue and cartilaginous areas were observed. Organs could not be distinguished. Placental cotyledons were observed completely. Eccentric insertion of the cord to the placenta was detected. Also 4 × 3 cm sub-chorionic bleeding area was detected in the placenta base (Figs. 3 and 4). Where fibroblasts were distinguished in the abdomen, microscopic evaluations revealed connective tissue, cartilage and fatty tissue. Sections belonging to skin, muscle and bone tissues were natural.

## Discussion

TRAP sequence is a rare phenomenon, particularly in monochorionic multiple pregnancies. It is caused by abnormal vascular connections between foetuses that are generally observed in the surface of the placenta and are sometimes formed in the umbilical cord. Arterial flow, which is expected to return to the placenta, passes through the pump foetus directly to the receiver foetus. When the hypoxic foetus receives even limited oxygen and food resources, it is possible for lower extremities to develop. However, as in this case, if hypoxia is deep and starts in the early weeks of gestation, organ development is not likely to occur. As hypoxia occurred in this case, Foetus C’s heart development was not completed; it could be seen as a non-functional single-ringed organ. The acardiac foetus is not viable, but the viability of the pump foetus depends on its cardiac function, which dictates its ability to feed both its own body and the acardiac foetal body.

While the follow-up and delivery of the case is explained in detail, there are some limitations while reporting. One of the limitations of the case is the inability of genetic examination of the acardiac foetus. Determining the chromosomal anomalies of the acardiac twin could help us to understand the genetic basis of TRAP sequance by comparing with case reports in the literature. The second limitation of this report is the insufficient microscopic determination of the tissues of acardiac foetus.

Detection of chromosomal abnormalities in the TRAP sequence may be useful. In previous studies, the ratio of chromosomal anomalies was 9% [3]. Monosomy, trisomy, polyploidy and deletions can be observed in chromosomal karyotyping of acardiac foetus [4]. Trisomy 2 in acardiac foetuses has been shown and other trisomies can be observed. [5].

Heart failure is very common in pump foetuses, and mortality rates can reach 50% [6]. Rare cases can withstand the third trimester without invasive manipulation [7, 8]. Identification of TRAP sequence cases in the early gestational weeks enables ligation and laser ablation.

Diagnosis of TRAP sequence cases is performed ultrasonographically [9]. The first phase in diagnosis is the detection of multiple anomalies in one of the foetuses in monocorionic multiple pregnancies. Acardiac and anencephaly are typical findings of foetus. Detection of reverse flow in acardiac foetus doppler flow velocity is a confirmatory finding. Ultrasonographic evaluations might indicate abnormal vascular connections in placenta or within umbilical cord. When these connections involve all foetuses, prognosis of pregnancy could become worse. While pathologic examination revealed anastomoses that connected Foetus B and C, no anastomoses were identified that belonged to Foetus A. Hence, fetal bradycardia was observed in Foetus B. Detection of polyhydroamniosis in the sac is due to the same reason.

Prognosis of the pump foetus after birth is associated with the degree of the effect of the fetal heart in the intrauterine life. When the diagnosis is made in early gestational weeks, the patient could be given the options of termination of pregnancy or selective fetal reduction. Selective reduction could be performed with the fetoscopic cord ligation of the acardiac foetus or with the ligation of abdominal aorta with radiofrequency. These interventions prolong the duration and the time needed until birth and could increase survival rates from 70 to 90% [10].

One of the factors that determine the prognosis of pregnancy is the size of acardiac foetus [11]. If the abdominal circumference of the acardiac foetus is equal to or bigger than the pump foetus; if doppler severe flow disorders has started in the pump foetus; if the pump foetus has developed hydrops findings and polyhydroamniosis; it means active management indication of pregnancy has risen. Radiofrequency ablation is the least invasive procedure. Invasive interventions in the cases diagnosed in the first trimester are considered to be more successful [12].

However, TRAP sequence cases in triplet pregnancies could be technically more difficult in comparison to twins. Venous connections’ affecting all three foetuses disturbs the hemodynamic balance. Given all these difficulties, conservative follow-up and treatment seem to be safer in monochorionic triplet pregnancies in comparison to invasive procedures.

Identification of trap sequence cases in early gestational weeks enables ligation and laser ablation. Fetal cardiac insufficiency, amount of amniotic fluid and fetal development discordance should be paid attention in patients who had late diagnosis. As the case in our study applied in the third trimester and as it was triplet pregnancy, she was followed with serial ultrasonography, doppler and fetal echocardiography. It is thought that individualization of TRAP cases in triplet pregnancies according to the conditions of the clinicians and fetal vein connections seems to be more convenient and beneficial.

## Conclusion

Identification of trap sequence cases allow for ligation and laser ablation in early gestational weeks. Clinicians should be careful in patients with fetal cardiac insufficiency, amount of amniotic fluid and fetal development discordance when patients had late diagnosis. As the case in our study applied in the third trimester and as it was triplet pregnancy, she was followed with serial ultrasonography, doppler and fetal echocardiography. It is thought that individualization of TRAP cases in triplet pregnancies according to the conditions of the clinicians and fetal vein connections seems to be more convenient and beneficial.

## Data Availability

Not applicable.

## Abbreviations

APGAR: Appearance, pulse, grimace, activity, respiration
BPM: Beats per minute
MoM: Multiple of median
MR: Magnetic resonance
TRAP: Twin reversed arterial perfusion
